# Living *hi*: GBMSM narratives of pleasure, risk, and everyday *hi-fun* (chemsex) navigation in Thailand

**DOI:** 10.1371/journal.pgph.0007177

**Published:** 2026-11-03

**Authors:** Worawalan Waratworawan, Nattharat Samoh, Alison J. Rodger, Harry Prabowo, Gloria Lai, Nittaya Phanuphak, Siripong Srichau, Verapun Ngamee, Adam Bourne, Thomas E. Guadamuz, T. Charles Witzel

**Affiliations:** 1 Institute for Global Health, University College London, London, United Kingdom; 2 Mahidol Center for Health, Behavior and Society, Faculty of Tropical Medicine, Mahidol University, Bangkok, Thailand; 3 Asia Pacific Network of People Living with HIV/AIDS, Bangkok, Thailand; 4 International Drug Policy Consortium, Bangkok, Thailand; 5 Institute of HIV Research and Innovation, Bangkok, Thailand; 6 Service Workers in Group Foundation (SWING), Bangkok, Thailand; 7 Ozone Foundation, Bangkok, Thailand; 8 Australian Research Centre in Sex, Health and Society, La Trobe University Melbourne, Victoria, Australia; University of the Philippines Diliman, PHILIPPINES

## Abstract

*Hi-fun* among gay, bisexual and other men who have sex with men (GBMSM) is practiced across Southeast Asia. Yet research remains focused on epidemiological trends and health risks, with limited qualitative work capturing lived experiences, motivations and support needs. These gaps are pronounced in Thailand, where social, legal and cultural structures emphasise control and enforced abstinence over harm reduction and holistic support. This study aims to explore the experiences and perspectives of GBMSM who engage in *hi-fun* and other types of sexualised drug use, including their strengths and challenges. It seeks to identify the types of support they desire to contribute to the development of harm reduction strategies grounded in the everyday contexts of GBMSM communities in Thailand. This qualitative study drew on five focus group discussions (FGDs) and six in-depth interviews (IDIs) with 30 Thai and non-Thai GBMSM in Bangkok, Khon Kaen and Pattaya. Discussions explored the contexts, experiences, strengths, challenges and support needs related to *hi-fun*. Thematic analysis was conducted, and findings were reviewed with a community advisory board and partner NGOs. Participants described *hi-fun* as a distinct world enabling self-expression, pleasure, intimacy and a sense of being valued and accepted beyond what everyday life affords. This world was shaped through equipment preparation, participant screening and unwritten rules promoting trust and safety. *Hi-fun* offered emotional and social benefits, including connection and belonging. However, participants also reported risks, including mental health challenges, dependence, sexual performance difficulties, violence, non-consensual recording, financial strain and legal consequences. *Hi-fun* became problematic when individuals lost control, encountered police, were forced into mandatory rehabilitation, experienced health decline or struggled with daily functioning. Participants emphasised the need for sterile equipment, post-use care, accurate information, stigma-free environments and legal reforms aligned with lived realities. Supporting GBMSM engaged in *hi-fun* in Thailand requires moving beyond abstinence-focused approaches toward flexible harm reduction and structural reforms that uphold dignity, safety and equity.

## Introduction

The phenomenon of *hi-fun* among gay, bisexual and other men who have sex with men (GBMSM) in Thailand, often called chemsex in Western countries, has gained increasing attention in academic, health system and media discussions [1–3]. *Hi-fun* refers to “the intentional combining of sex and crystal methamphetamine (*ice*) to enhance intimacy and pleasure with one or more other man/men, facilitated by technology and usually in a private setting” [4]. These practices often occur in informal but prearranged contexts organised through dating apps and may involve multi-day sex parties. Previous ethnographic research has highlighted that *hi-fun* scenes extend beyond drug use itself and are shaped by the physical, social, and atmospheric qualities of private settings, including lighting, music, and interpersonal interactions that contribute to experiences of intimacy, pleasure, and secrecy [1]. Many men who engage in *hi-fun* intentionally use substances to increase confidence, disinhibition, and openness to more intense sexual experiences [5].

Existing research has helped identify several health implications of *hi-fun*, including a heightened risk of acquiring HIV and sexually transmitted infections (STIs), sexual violence and mental health concerns, and has highlighted the need for health-promoting activities for GBMSM who use substances [6–9]. Emerging qualitative studies from Southeast Asia have also demonstrated that *hi-fun* is experienced as more than a public health risk. Across Thailand, Singapore, and Malaysia, qualitative research has described *hi-fun* as a social and relational practice through which participants pursue intimacy, sexual pleasure, emotional connection, and a sense of belonging while simultaneously navigating stigma, criminalisation, and barriers to appropriate health services. These studies also highlight the importance of peer networks and community-based support in sharing practical knowledge and harm reduction strategies [1,5,10,11]. Despite these emerging qualitative insights, much of the broader literature continues to rely on risk-based framings and pays limited attention to the social, emotional, and cultural meanings attached to *hi-fun* [12–14]. Although some studies in high-income countries have begun to explore pleasure, agency, and lived experience in sexualised drug use [13,15,16], such research remains relatively limited in low- and middle-income settings. In Southeast Asia, qualitative studies are beginning to emerge, yet the voices of people with lived experience remain underrepresented. This constrains the development of interventions that acknowledge the pleasure and value derived from *hi-fun*, factors shown to support more engaging and effective service provision [17].

Exploring *hi-fun* in Thailand is further complicated by the sensitivity of drug use, sexuality, and sexual diversity in public discourse. When these topics are discussed publicly, they are often framed moralistically and stigmatised, reinforced by criminalisation, compulsory treatment systems and narratives that position drug use as deviance rather than a health issue [18,19].

In Thailand, family and social expectations rooted in patriarchal and patrilineal values add further pressure. In Thai Chinese families, sons are regarded as responsible for maintaining male lineage, while daughters marry out of the family [20,21]. These expectations, strengthened by notions of filial piety, place heavy burdens on sons to meet familial and social obligations [1,22–25]. As a result, many GBMSM live under intense pressure from families, health systems, and state policies that leave little room for personal desires, pleasure, or self-defined ways of managing life.

In policy settings, *hi-fun* is often reduced to a biomedical health issue, overshadowing social, cultural, and community contexts, as well as the benefits individuals describe in their engagement with sexualised drug use [26]. Although the United Nations Office on Drugs and Crime has urged member states to shift from punitive drug approaches toward models grounded in human rights [27], the Thai legal system has yet to fully realise such reforms. Persistently fragmented and inconsistent legislation creates barriers for marginalised groups. For example, repeated revisions to the ministerial regulation specifying methamphetamine possession thresholds under the Thai Narcotics Code have created uncertainty in both legal interpretation and enforcement. Lower thresholds increase the likelihood that individuals possessing drugs for personal use are misclassified as traffickers, as the legal thresholds do not necessarily reflect real-world consumption patterns [28–30]. This ambiguity heightens legal risk for *hi-fun* participants and discourages them from seeking healthcare or support. Methamphetamine and similar substances remain heavily criminalised, further contributing to reluctance in accessing services [9].

Biomedical paradigms shaping current knowledge surrounding *hi-fun* tend to emphasise treatment or harm reduction by positioning users as patients in need of management rather than respecting them as people with narratives, desires, and their own strategies for living. Viewed through the lens of Critical Medical Anthropology [31], these structures often silence vulnerable groups through forms of structural violence including social exclusion, stigma, and unequal access to resources [32]. Framing *hi-fun* primarily in terms of disease and risk reduces its complexity and allows dominant systems to reinforce definitions of abnormality. Although harm reduction has gained increasing recognition, drug-related discourse continues to be shaped by medicalised frameworks that often separate drug use from its social, cultural and structural contexts [33,34]. Without listening to people with lived experience, support systems risk reinforcing power imbalances and failing to uphold dignity and human needs [31].

This study seeks to create meaningful space for the authentic voices of people with *hi-fun* experience. It explores how individuals define sexualised drug use and interpret its pleasures, risks and vulnerabilities, and identifies the kinds of support that best align with their lived contexts. We draw on the diverse settings of Bangkok, Khon Kaen, and Pattaya, recognising that no single narrative captures the experiences of all participants and avoiding reducing their accounts to external or dominant frameworks [35]. By grounding analysis in lived realities, the research outlines supportive approaches including harm reduction strategies that uphold physical safety along with dignity, agency, and human needs.

This qualitative study therefore aims to explore the experiences and perspectives of GBMSM who engage in *hi-fun* and other types of sexualised drug use, including their strengths and challenges. It seeks to identify the types of support they desire in order to contribute to the development of harm reduction strategies grounded in the everyday contexts of GBMSM communities in Thailand.

## Methods

### Ethics statement

Ethical approval for this study was obtained from the Research Ethics Committee, Faculty of Social Sciences and Humanities, Mahidol University (COA 2024/030.1902) and the University College London Research Ethics Committee (COA 24583/001). All participants were aged 18 years or older. Verbal informed consent was obtained from all participants prior to participation in the study, including consent for audio recording of the interviews. To protect participants’ confidentiality, each participant was assigned a unique study code, which was used in place of their name during the interviews and throughout the research process.

### Study design

We conducted a qualitative study using focus group discussions (FGDs) to explore community norms, supplemented by in-depth interviews (IDIs) to add depth and capture the perspectives of GBMSM with privacy concerns.

### Coproduction

The study was co-produced with a Community Advisory Board (CAB) established for this research. The CAB was an independent advisory group rather than a formal governing body; the study itself was conducted by public universities. The CAB comprised representatives from local and international non-governmental organisations (NGOs), clinicians, organisations working in HIV and harm reduction, regional networks of people living with HIV and people who use drugs. Several members also represented organisations engaged in harm reduction policy advocacy in Thailand. The CAB shaped the selection of study sites, provided ongoing guidance during data collection, contributed to the development of data collection instruments, recruitment strategies, and the analysis and interpretation of findings. This helped ensure that the research remained grounded in community priorities and lived experience.

### Selection and recruitment

Preparatory coordination with partner NGOs took place between March and April 2024. Participant recruitment and data collection were conducted between 18 May and 31 August 2024 through NGOs experienced in working with GBMSM in three locations: Bangkok (with the Institute of HIV Research and Innovation), Khon Kaen (with ACTTEAM) and Pattaya (with the Health and Opportunity Network). Additional participants were recruited through investigators’ social networks. Eligible participants were cisgender or transgender men aged 18 or older who reported using one or more of crystal methamphetamine, GHB/GBL, ketamine, MDMA, cocaine or other stimulants before or during sex with another man within the past 12 months. This eligibility criterion was intentionally adopted to capture the diversity of substance use practices in sexualised settings rather than restricting recruitment to individuals using crystal methamphetamine alone. Although no sampling frame was used, we required that at least 20% of the sample be born outside Thailand to reflect the diversity of GBMSM communities in Thailand.

The three study sites were selected following consultations with the CAB and community-based organisations with extensive experience working with GBMSM and people who use drugs across Thailand. These consultations informed the selection of sites with active community-based organisations and distinct social and cultural contexts relevant to *hi-fun*. Bangkok represents an urban centre attracting Thai and foreign GBMSM. Khon Kaen reflects a regional multicultural setting with diverse populations, including students, workers and migrant labourers. Pattaya is a tourism-driven city linked to the service industry and transactional sex. Together, these varied environments capture diverse GBMSM experiences across Thailand.

### Data collection

Topic guides for focus groups and interviews addressed issues related to sexualised drug use including *hi-fun*, motivations, challenges and support needs [4]. Several days before data collection, NGO staff gave participants information about the study aims, procedures, confidentiality and their right to withdraw. Additional clarification was provided as needed.

Verbal informed consent was obtained and audio recorded. Researchers explained the study purpose, risks and data protection measures before beginning each session. To minimise stigma and discomfort, anonymity was prioritised: no names were collected unless required for recruitment, and any that were collected were promptly destroyed. A short anonymous demographic survey captured age, gender identity, education, HIV status, PrEP use, substances used during sex and country of birth.

During data generation, participants were referred to by numerical codes and reminded not to use names when discussing experiences. They were invited to reflect on strengths and challenges of *hi-fun* and identify priority issues. Data collection was conducted by three trained researchers with expertise in ethics and qualitative interviewing (WW, NS & TCW). Sessions were held at NGO or university offices. Participants received THB 1,000 as compensation.

### Data analysis

Audio files were transcribed verbatim, translated where necessary, reviewed and anonymised. Thematic analysis followed established procedures [36]. WW and TCW reviewed the transcripts to develop preliminary themes, which were then tested and refined during coding. New themes were incorporated when researchers reached consensus. Two researchers independently coded all transcripts using NVivo, and all data were managed within the UCL Data Safe Haven. The research team subsequently consolidated the key themes and shared preliminary findings with the advisory board and NGO partners for feedback and triangulation.

## Results

We conducted five FGDs and six IDIs with 30 participants across Bangkok (n = 12), Khon Kaen (n = 9), and Pattaya (n = 9). FGDs had between two and nine participants each. Most participants identified as gay (83%, n = 25) and had completed at least a bachelor’s degree (60%, n = 18). Nearly all were aged 26–45 (93%, n = 28). One third were living with HIV; among participants not living with HIV, nine were using PrEP. Crystal methamphetamine was the most commonly used substance in the past 12 months (n = 23), followed by MDMA (n = 14), ketamine (n = 12), cocaine (n = 10) and GHB/GBL (n = 5). Most were born in Thailand (77%, n = 23), and seven (23%) were born elsewhere. See Table 1 for full demographics.

**Table 1:** Participant demographics.

Characteristic	N = 30
Age	
18–25	1
26–35	16
36–45	12
46+	1
Sexual orientation	
Gay	25
Bisexual	5
Country of birth	
Born in Thailand	23
Born outside Thailand	7
HIV status	
Negative	6
Negative/taking PrEP	9
Living with HIV	10
Prefer not to say	5
Highest education	
Bachelor’s degree or higher	18
Secondary school	3
Below secondary school	9
Location	
Bangkok	12
Khon Kaen	9
Pattaya	9
Drugs used in past 12 months	
Cocaine	10
Ecstasy/MDMA	14
GHB/GBL	5
Ketamine	12
Crystal methamphetamine	23

Findings are presented in six themes: (1) setting the scene: inside the world of *hi-fun*; (2) pleasure, community and identity: *hi-fun* strengths; (3) behind the *hi*: experiences, concerns and risks participants encounter; (4) beyond control: when *hi-fun* becomes a problem; (5) walking the line: managing use and minimising harm; and (6) what we need: support beyond abstinence.

### Setting the scene: Inside the world of *hi-fun*

Over half the participants described *hi-fun* as entering a distinct world—a space separate from everyday life where they could express themselves freely and experience joy, authenticity and escape. Rather than being impulsive or chaotic, this world was intentionally created through careful preparation. Participants described obtaining substances, especially *ice* (crystal methamphetamine), along with tools such as syringes, saline solution and sexual-enhancement products. In Bangkok, access to items like saline was limited, sometimes leading to higher resale prices, particularly at night.

Sessions often lasted two to three days, typically on weekends or holidays. Participants stocked snacks and sugary drinks and preferred not to leave the space once inside, reinforcing the sense of a contained *hi-fun* world designed for continuity and immersion.

**Participant 7:** *“Yeah, you need to prepare drinks and stuff. Can’t eat rice, gotta eat congee. The fridge has to be stocked. No going out.”*

Thai language focus group, Pattaya

**Participant 4:** *“From what I heard, you need to have spare needles—at least three or four per person. Some people might keep around ten. You also need saline, which is hard to find, especially at night. And since it’s in demand, some people resell it at a higher price”*

Thai language focus group, Bangkok

Selecting the right people was central to shaping this world. Participants screened potential partners to manage risks such as undercover policing, theft or incompatible dynamics. Screening through photos or video of the face, body or genitals allowed participants to assess attraction, compatibility, perceived safety and mutual sexual interest. These appearance-based evaluations frequently determined who was considered suitable to join a session. When compatibility was strong, the world of *hi-fun* felt pleasurable and emotionally engaging; when it was not, participants often withdrew to avoid disrupting the experience.

**Participant 1:** *“I set up a meet-up with this guy, and he tells me he has a friend with him. But I don’t know what this friend looks like, and he won’t let me see. But the guy I was talking to—he looked good, really good, so I was like, okay, just the two of us is fine. But then, when I got there, the other guy was... not my type. And like, I wanted to play with the hot guy, but when the other dude realized I wasn’t into him, he got moody. And then that made me feel awkward too. So I was like, nope, not doing this. It wasn’t really my fault, but at the same time, I kinda killed the mood.”*

**Participant 3:** *“Seeing the face first”*

Thai language focus group, Bangkok

Atmosphere was another deliberate part of constructing the *hi-fun* world. Participants shaped lighting, sound and environment to cultivate mood, intimacy and safety. Soft or dim lighting was preferred for privacy and confidence. Some used music or television to sustain arousal, while others preferred silence to avoid attention, especially in hotels. In Khon Kaen, participants often used resorts or hotels because hosting at home risked exposure in tight-knit communities. Across settings, managing the sensory environment was essential to sustaining the protected world participants created.

**Participant 5:** *“Yeah, you can play music without worrying about the neighbors.”*

**Participant 2:** *“If it’s in a private condo, sure. But not in a hotel.”*

**Participant 3:** *“In a hotel, we might just leave the TV on.”*

**Participant 2:** *“Or play porn.”*

Thai language focus group, Khon Kaen

Participants also described unwritten rules that governed behaviour and maintained trust and cohesion. Leaving early could raise suspicion, and phone use was discouraged because it disrupted emotional connection and could be perceived as a security threat. Shared expectations around substance use also reinforced collective participation: many emphasised that *hi-fun* was a shared experience and that using the same substances in the same ways contributed to the sense of being in a unified, mutually dependent world.

**Participant 8:** *“But most people don’t use their phones because others might get suspicious—like, who are they talking to? Are they a police informant? So they just put their phones away and focus on what they’re doing.” “I don’t play with people who use their phones. I tell them straight up—put it away. If you need to check your phone, you have to ask first.”*

Thai language focus group, Pattaya

### Pleasure, community, and identity: *Hi-fun* strengths

Many participants described *hi-fun* as offering more than sexual pleasure. It functioned as an emotional and psychological space of release where they could reduce stress, ease loneliness and express themselves without judgement, which was particularly important in social contexts where sexual minorities often feel marginalised. Using *ice* heightened excitement, prolonged sexual pleasure and created experiences that felt distinct from everyday encounters. In Bangkok, participants highlighted the intensity of sex and how substances deepened emotional connection. In Pattaya, *hi-fun* was also seen as a way to cope with work stress and pressures tied to the tourism and sex-work economy. Across sites, *hi-fun* emerged as a space where participants could experience emotional relief and express aspects of themselves that were often constrained in everyday life. Participants described temporarily leaving behind work pressures and daily worries while experiencing emotional connection with others. Many also described feeling desired, chosen, and affirmed in ways rarely experienced in their everyday lives.

**Participant 1:** *“It helps to leave all the life problems behind and focus on our own world.”*

**Participant 8:** *“It’s like it cuts off the problems we’re facing and reduces the worries.”*

**Participant 4:** *“It takes us to think about something else instead of what we’re worried about.”*

Thai language focus group, Pattaya

**Participant 3:** “I like that I can truly be myself when I’m with my friends.”

Thai language focus group, Khon Kaen

Some participants also developed trusted friendships that extended beyond *hi-fun* sessions into their everyday lives. These relationships provided emotional and practical support, fostered a sense of belonging, and increased confidence in choosing supportive relationships.

*“I found good friends who support my business and are there when I have problems. We can talk about everything. I also met them through hi-fun. I cut off the people who weren’t good for me… For me, this community comes with happiness. If you find the right people here, you can be genuinely happy.”*

Thai language in-depth interview, Khon Kaen

Some participants described gaining skills through *hi-fun* participation. These included safer injecting practices, caring for friends in cases of overdose, and learning how to manage drug interactions and bodily responses. Regular injectors developed expertise in preparing equipment, choosing injection sites and administering basic first aid. Such knowledge reflects a form of community resilience built within *hi-fun* spaces.

**Participant 8:** *“It’s like being a field medic. Some people get so skilled that they even inject for others. Sometimes, I don’t even want to play, but they’ll ask, ‘Can you inject me?’ So I do it, and then they stay while I leave. They trust me because we’ve known each other for a long time. But I’d never inject for someone I don’t know.”*

**Participant 8:** *“I even took a basic first aid course so I understand how to handle these situations.”*

Thai language focus group, Pattaya

Despite these skills, mutual care was not consistently present. Only a few participants recalled receiving support during early drug use or emergencies. In Khon Kaen, *hi-fun* was described as intimate and personally meaningful, but opportunities for group care were limited. These accounts reveal that while *hi-fun* can foster self-reliance and occasional networks of care, access to such support varies considerably across groups.

**Participant 5:** *“Taking care of each other is not something we often see.”*

**Participant all:** *“It’s very rare, I only see it the first time.”*

Thai language focus group, Khon Kaen

### Behind the *hi*: Experiences, concerns and risks participants encounter

Many participants described mental health challenges as one of the most significant but least discussed difficulties linked to *hi-fun*. Experiences such as loss of control, paranoia, hallucinations, impaired judgement and emotional withdrawal were common, alongside intense comedowns that sometimes triggered deep distress. In Pattaya, these emotional crashes were often described as intertwined with romantic or relationship difficulties and, for some, led to thoughts of self-harm.

**Participant 8:** *“Yeah, for some people, it depends on whether they can control themselves or not. I once tried to take my own life—I took Pet-Pro* [toilet bleach]*. At that moment, I felt like I had nothing left. I had just broken up with my long-term partner, and my mind was racing with all these thoughts. I wasn’t even thinking about using or not using anymore—I just felt completely empty. It was the darkest feeling, like I was done with life. I was getting older, we had been together for so long, and if they were going to be with someone else, that was it. That was the end. It all happened in just a split second, but that’s how deep the comedown hit me.”*

Thai language focus group, Pattaya

Some participants also expressed concerns that sex without substances no longer felt satisfying after experiencing the heightened pleasure, confidence and intimacy associated with *hi-fun*. Some felt their sexual identity and fulfilment became dependent on drug-enhanced encounters.

Erectile dysfunction was another prominent concern, especially among frequent users. Because sex was central to *hi-fun*, difficulties maintaining an erection could disrupt the session and generate anxiety, leading many to rely on medications such as sildenafil (Viagra) to sustain performance. These experiences shaped feelings of confidence, self-image and the overall quality of *hi-fun* encounters.

**Participant 5:** *“One of the biggest problems during a session is when the dick won’t get hard. It’s the main issue. Whether you’re the top or whatever—if it’s not hard, the bottom just has to sit there waiting, wondering, ‘Will it even happen?’ And by the time it does, the mood’s already gone.”*

**Participant 5:** *“It’s exhausting. I tried helping him all night and still nothing. I was so tired.”*

**Participant 6:** *“I spent the whole night trying to build him up, and it just wouldn’t rise.”*

Thai language focus group, Khon Kaen

Participants also worried about physical and sexual health risks, including potential exposure to STIs, fatigue, insomnia and weight loss. Some consequences appeared immediately after use; others emerged with prolonged engagement. Although discussed less often, overdose, cardiac issues and injecting-related harms were acknowledged as concerns.

**Participant 7:** *“We never really know. You just have to be mentally prepared. It’s not like you can stop and ask, ‘Hey, do you have anything? Are you infected?’ No one cares about that in the moment—at that point, it just doesn’t matter anymore.”*

Thai language focus group, Pattaya

**Participant 2:** *“My taste perception has changed—I can’t handle spicy food anymore.”*

**Participant 3:** *“It only affects eating during the first couple of days after using. After two or three days, my tongue and everything go back to normal. But right after a session, I can’t eat anything—it feels like my tongue and throat are sore.”*

Thai language focus group, Khon Kaen

Legal risks added another layer of fear. Because drug use in Thailand carries criminal penalties and can result in mandatory rehabilitation or incarceration, many participants experienced ongoing paranoia about police inspections, raids or undercover officers. Some described being searched, arrested or pressured to pay bribes. These fears made *hi-fun* feel unpredictable and emotionally taxing.

**Participant 3:** *“It really affects you—when you’re deep into it, you start getting paranoid. Seeing lights, anything at all, and you think it’s the police. Even if there are no cops around, sometimes you still freak out.”*

**Participant 3:** *“Fear of the police is the number one thing. No one wants to get arrested.”*

Thai language focus group, Bangkok

Safety concerns extended beyond law enforcement. Participants described risks of violence, theft, deception and non-consensual recording, particularly when meeting unfamiliar partners. The unpredictability of others’ behaviour contributed to a sense of vulnerability, and some men prepared strategies in advance to manage conflicts or unexpected incidents.

**Participant 6:** *“Yeah, it happens. Some people are into sadism.”*

**Participant 6:** *“Slapping, hair-pulling.”*

**Participant 4:** *“I’ve only experienced getting peed on.”*

**Participant 2:** *“Physical violence.”*

**Participant 2:** *“Losing control of emotions.”*

**Participant 2:** *“Got robbed, got scammed.”*

Thai language focus group, Khon Kaen

*“Violence happens because people get emotionally unstable. If someone isn’t satisfied after using, they might scratch or even attack. I’ve heard stories of people getting scratched, leaving marks, and showing them later—it happens.”*

Thai language in-depth interview, Bangkok

Finally, participants noted the financial strain associated with *hi-fun*, including costs for drugs, accommodation, equipment and transport. Some turned to additional income sources, gambling or pawning belongings in order to continue participating.

**Participant 1:** *“Losing money, rising expenses, struggling to keep up with cash flow—because I have to buy stuff to play.”*

**Participant 3:** *“Spinning slots to make money.”*

**Participant 4:** *“Pawned my motorbike.”*

Thai language focus group, Khon Kaen

### Beyond control: When *hi-fun* becomes a problem

In addition to the risks linked to *hi-fun*, participants described several situations in which *hi-fun* shifted from pleasurable to problematic. A common indicator was an increase in frequency beyond one’s personal limits. For many, engaging more often than intended signalled a loss of control and raised concerns about dependence.

*“For others, I’m not sure because our partner has a problem with the night. They...they have no limit. Sometimes they contact for three days, then they sleep, then they sleep for one night, then they can continue. They’ll sleep for about three days in a row. Sometimes they’re out of it for a week before they continue. I think their factor is money. If they have money, they’ll forget immediately, keep going immediately. For us, we have a usage limit, right? It has to be this much, enough. It’s enforced by ourselves. We can’t break our own enforcement rule because we fear of 1. becoming mentally addicted to drugs and becoming so addicted that we can’t live our daily lives.”*

Thai language in-depth interview, Khon Kaen

Loss of control extended beyond frequency. In Bangkok, participants described difficulties regulating their behaviour during sessions, including distorted perceptions, risky interactions or actions they would not normally take. When these behaviours emerged, men began to view their *hi-fun* use as problematic.

**Participant 1:** *“He mentioned that while they were engaged in an activity, he asked someone to block the pipe in the bathroom. So, we were confused; why was he afraid of cockroaches? Then he said someone would come out through the pipe. And I’m thinking, what kind of person can come out through a pipe?”*

Thai language focus group, Bangkok

Being unable to recall events during *hi-fun* was another sign of concern. Participants also noted that social withdrawal, reduced interaction with others and declining work performance indicated that *hi-fun* was negatively affecting their daily lives. Participants also linked these consequences to financial strain and deteriorating physical health.

**Participant 3:** *“Issues with performance at work or you’ve been ill a lot or things like that which then would be more financial.”*

English language focus group, Bangkok

Social harms were also viewed as key indicators of problematic *hi-fun*. In Pattaya, men frequently described experiences of violence, coercion and being pressured into situations against their will, including interactions with police. Legal involvement, particularly arrest or mandated rehabilitation, was widely understood as a sign that *hi-fun* had escalated into a serious problem.

In addition, some participants recounted incidents of sexual violence, unwanted recording, or being forced to take unknown substances. These events were described as occurring when control was lost, either personally or within the group, and were considered clear markers that *hi-fun* had become unsafe.

**Participant 4:** *“They were so kinky, like taking their penises out and pissing on each other. That really disturbed me, I was so scared. I didn’t dare to do anything at all. I was afraid of contagious diseases and needles. I didn’t know whose needle it was. But there were things I had never seen before, like taking off pants, pissing on each other, drinking piss, and stuff like that. It was very scary. I couldn’t handle it. I was afraid.”*

Thai language focus group, Pattaya

**Participant 3:** *“Those experiences with physical assault, theft, secret filming, and all these kinds of things.”*

Thai language focus group, Bangkok

### Walking the line: Managing use and minimising harm

Although participants recognised the risks associated with *hi-fun*, many described strategies to manage and reduce harm. Some viewed substances as harmful but manageable when used with clear limits. Participants described acquiring practical knowledge about drug use informally through partners and more experienced members of the *hi-fun* community. This included avoiding certain drug combinations, such as using poppers simultaneously with *ice*, because of the perceived risk of cardiac complications. Many also set personal rules around frequency, often restricting *hi-fun* to once a month to prevent dependence, maintain pleasure and protect their health.

**Participant 3:** *“In some groups, people use* ice *first and only inhale poppers afterwards because using them at the same time can overstimulate the heart and make it beat too fast.”*

**Participant 2:** *“For some people, their heart rate becomes so fast that they can go into shock or overdose.”*

Thai language focus group, Khon Kaen

*“Yeah, could I do it more often? Of course. But I set limits for myself. I worry about my body—looking worn out, aging faster. That’s why I restrict myself. Could I keep going if I wanted to? Sure. But I have to tell myself to stop. Enough is enough.”*

Thai language in-depth interview, Khon Kaen

Building on this shared knowledge, participants also learned how to smoke and inject drugs, and prepared sterile equipment in advance, bringing multiple spare needles and saline to avoid having to share equipment during sessions.

**Participant 8:** *“The first person who introduced me to* ice *was my boyfriend… At first, I didn’t know how to smoke it… He said, ‘Just try it.’… After we broke up, I was introduced to injecting by someone else… That person taught me, saying, ‘This is how you do it.’… Now I know how to do it myself… I’ve become like a doctor.”*

Thai language focus group, Pattaya

**Participant 4:** *“…You have to bring spare needles. For me, I usually prepare three or four at minimum… If there are several people, I might bring around ten… I also bring saline.”*

**Participant 3: “**…When I buy drugs, they include needles…I already have cotton and alcohol at home.”

Thai language focus group, Bangkok

Other strategies included keeping contact information for harm reduction NGOs and informing trusted friends about session details in case of emergencies. These practices demonstrate how individuals actively managed risks and protected themselves and others. Legal risk management was another important concern. In Bangkok, some men carried NGO-issued identification or health documents to present during police stops. However, most emphasised avoiding possession of substances, especially at night when inspections were more common. Others described asking female friends to carry drugs because they were less likely to be searched. In Khon Kaen, strategies focused more on maintaining health, such as limiting sessions and monitoring the physical impacts of use. A few European participants believed they could pay bribes if confronted by police. These diverse strategies highlight that individuals were not passive within structural constraints but actively navigated them through personal and pragmatic adaptations.

**Participant 1:** *“For me, I can show you so you get a clearer picture—it’s harm reduction. This one is from* [redacted organization]*. Honestly, I’m not actually in rehab, but I know about it because I used to work with* [redacted organization]*. Having harm reduction tools can help protect us when we go out and face drug checkpoints. The key thing is: don’t carry anything on you. If your urine test comes back positive, that’s fine. Let them test. Once they see the result, just show them this document. They’ll check with the organization to confirm you’re in treatment. I need to get a new one, though, since it’s been a few months.”*

Thai language focus group, Bangkok

### What we need: Support beyond abstinence

Participants emphasised that their needs extended beyond abstinence-based support. They highlighted the importance of resources that enable safer use and promote physical and emotional recovery. In Khon Kaen, men described needing vitamins, herbal detox remedies and post-use care, along with access to clean equipment and reliable information about safer practices. Some recounted having to reuse needles or clean them with water, saline or alcohol when supplies were unavailable, which they viewed as risky. These accounts reflect a harm reduction approach in which people seek tools and knowledge to care for their bodies in realistic conditions rather than through abstinence alone.

**Participant 2:** *“I’d love better access to things like free vitamin C.”*

**Participant 2:** *“For people who play, there should be something to help flush out toxins after using. There are detox herbs, like* Rang Jeud *(Thunbergia laurifolia) and things like that.”*

**Participant 4:** *“When I went to see a doctor, they gave me about a week’s supply—morning and evening doses.”*

Thai language focus group, Khon Kaen

**Participants 1&2:** *“Public health services should provide clean equipment. Let’s be real—people won’t stop using. So, instead of trying to make them quit, just make it safer. If they’re going to play, at least let them do it in a clean and safe way.”*

Thai language focus group, Bangkok

Participants also stressed the need for accurate and accessible information on safer drug use. They wanted practical guidance on mixing substances, safe injecting techniques and understanding drug interactions. While many actively sought to avoid harm, they often lacked non-judgemental and trustworthy sources of information. Existing abstinence-focused approaches were described as inadequate for addressing the realities of substance use.

**Participant 4:** *“I wish people had better education on how to use safely.”*

**Participant 4:** *“Yeah, it needs to be more accessible.”*

**Participant 4:** *“The problem is, people get suspicious—like, ‘Why are you here? What do you see me as?’ They don’t think about the fact that some people mix with plain water, while others use saline solution for nasal rinsing.”*

**Participant 4:** *“Yeah, some people don’t have needles and end up reusing old ones.”*

**Participant 2:** *“There’s still a lack of knowledge about harm reduction. Like, I’ve seen cases where people run out of their own needles, pharmacies are closed*

**Participant 3:** *“They have no choice but to reuse.”*

**Participant 5:** *“They keep using it until the needle gets dull.”*

**Participant 6:** *“Some just rinse it with water, others use saline, and some even soak it in alcohol before using it again. Everyone has their own way.”*

Thai language focus group, Khon Kaen

Beyond physical resources and knowledge, participants repeatedly highlighted the importance of acceptance and understanding from families, communities and society. Supportive, non-stigmatising environments were described as strengthening their ability to live well and manage use more safely.

Participants also expressed a need for broader structural change. They especially emphasised reform of legal and policing practices, noting that bribery, harassment and inconsistent enforcement created profound instability in their lives. Some men reported being fined or extorted even when they were not carrying substances but merely tested positive for drugs during forced testing. They described legal thresholds for drug possession as unclear and misaligned with everyday patterns of use, contributing to fear and vulnerability.

These calls are not merely policy suggestions; they reflect a deeper demand for structurally safe spaces where individuals who use substances can care for themselves, access support, and live with dignity, without fear of prosecution, exploitation, or systemic abuse.

**Participant 4:** *“Yeah, the regulations on drug quantity aren’t clear. Like, the allowed amount is really small—maybe just 0.1 of something. If you compare it to what’s in a needle, it might be around 0.5 or even less, which is barely enough to carry or inject. But if it’s 1 gram, that’s considered a lot. And there’s still no clear distinction between being a user and a dealer based on quantity. The weight also varies depending on how it’s packaged, so the criteria aren’t consistent.”*

**Participant 1:** *“With such a small amount, you’re immediately classified as a dealer.”*

**Participant 4: “**[I’d like them to*] set clearer size and quantity limits for possession.”*

Thai language focus group, Bangkok

## Discussion

In this study of 30 GBMSM with experience of sexualised drug use, *hi-fun* emerged as more than a setting for drug-enhanced sex. Participants described it as a deliberately constructed social space that provided acceptance, emotional connection, opportunities for self-expression, and relief from everyday stress and loneliness. At the same time, *hi-fun* involved community-developed safety practices and remained shaped by ongoing tensions between pleasure, risk and control.

Rather than being simply the ‘risky’ behaviour defined by the state [37], *hi-fun* was experienced as a meaningful social world that coexisted with concerns about substance dependence, sexual performance, and physical and mental health. Importantly, participants distinguished manageable risks from situations in which control was lost, highlighting the need for safer-use resources, post-use care, and legal reforms that better reflect the realities of substance use.

Indeed, *hi-fun* became problematic precisely when control was lost. This included difficulties in regulating frequency of use, sustaining daily functioning and work performance, or facing external forces such as police, the law, and compulsory rehabilitation. Arrest and judicial processes were therefore described as clear markers that *hi-fun* had become a problem. Similarly, situations beyond participants’ control, such as being subjected to violence, being secretly recorded, being coerced into unwanted sexual acts or excessive substance use, or being unable to recall what had happened during a session, were all cited as markers that *hi-fun* had become problematic. From these accounts, participants emphasised their desire for access to resources that would allow them to use substances more safely, to receive appropriate post-use care, and to gain knowledge relevant to their community. Most importantly, they expressed the need for legal reforms that recognise and respond to the realities of substance use, reflecting their call for a safer and more just environment in which their experiences could be understood and respected.

As demonstrated in this study of GBMSM’s experiences with *hi-fun* across three urban contexts in Thailand, *hi-fun* is not merely a site for drug-enhanced sexual activity, but a deliberately constructed world. It is designed to offer safety, privacy, and separation from mainstream societal scrutiny and normative moral frameworks. These spaces allow participants to express fluid identities, be themselves, and access forms of pleasure often denied in everyday life. This aligns with Bryant et al. [15], who argue that sexual pleasure is a critical yet overlooked aspect within traditional health frameworks in Western chemsex research. However, this study extends those understandings by showing how pleasure in Thailand’s *hi-fun* contexts is deeply intertwined with survival and the pursuit of legitimacy as citizens in the face of punitive legal systems and moral stigma. *Hi-fun* is not a boundlessly free space but one governed by unwritten rules and structured group discourses that form a distinct “scene” with its own logic [38,39].

Participants’ accounts suggest that becoming part of this world involved an ongoing process of socialisation in which practical knowledge and community norms were learned through interpersonal relationships and repeated participation. Men described learning how to prepare injecting equipment, manage drug interactions, recognise overdose, administer basic first aid, and navigate legal risks, while simultaneously acquiring the social expectations of *hi-fun*, including preparing the physical environment, screening participants, maintaining trust, and adhering to unwritten behavioural norms. Some participants even described becoming “like a doctor” or a “field medic,” illustrating how expertise, care, and responsibility were cultivated within *hi-fun* networks. These shared practices shaped not only how *hi-fun* sessions were organised but also who was recognised as a trusted and appropriate participant. Selection criteria based on appearance and perceived bodily desirability further demonstrate how mainstream norms around beauty and masculinity were reproduced within these seemingly countercultural settings.

Behaviour in *hi-fun* is further regulated by informal rules aimed at fostering participation, such as bans on phone use during sessions or expectations that everyone would participate actively in sex and substance use. These norms powerfully shape experiences and define who is considered an insider or an outsider. This reflects Michel Foucault’s concept of heterotopia, a space outside normative life that mirrors, resists or generates experiences impossible elsewhere, yet remains materially and symbolically powerful. *Hi-fun* appears liberating but operates through subtle mechanisms of ordering, selection, and discursive control embedded in participants’ routines [40,41]. This is strongly supported by Nagington [16], who challenges normative moral frameworks and describes chemsex scenes as subcultures with their own moral orders and internal regulations. Power emerges not from external authorities but from mutual surveillance, adaptation, and collective consent among participants [16].

Within these group-specific structures, *hi-fun* participants express diverse selves, not as pre-existing identities but as selves brought into being through performance and repetition [42]. These performances include emotional engagement in activities, proactive roles for receptive partners (e.g., stimulating partners’ genitals), and creating a sexy, fun atmosphere aligned with *hi-fun* community norms. Through these repeated performances, participants described feeling affirmed, desired, and emotionally fulfilled in ways that were rarely accessible in everyday life. Viewed through Butler’s concept of performativity, these experiences suggest that *hi-fun* could function as a space where participants expressed and affirmed aspects of themselves, while also fostering connection and emotional relief. This finding aligns with emerging critical chemsex scholarship, which encourages understanding sexualised drug use beyond the risk paradigm by recognising its roles in pleasure, intimacy, affect, and sociality [13]. Consistent with this perspective, participants described *hi-fun* as helping them cope with everyday stress and loneliness and giving them a sense of belonging, while also recognising the health and social risks associated with these practices [43].

In the face of risk and adversity, participants were not passive victims or powerless marginal subjects. Rather, they actively negotiated and managed risk, practised self-care, and strategically mobilised social, cultural, and bodily capital in response to structural constraints. These include unclear laws, moral stigma, and exclusion even within gay communities, where people who use drugs are often othered and cast outside the respectable mainstream. They are further constrained by biomedical frameworks that categorise them as “patients” or “deviants” [44,45].

Participants are thus not merely shaped by structure but act as agents who contest it. They create space, generate meaning, and sustain norms through practice, decisions, and relationship networks. These actions go beyond escaping structural violence [46]; they are claims to visibility and citizenship. This directly challenges biomedical discourses that reduce people who use drugs to health problems requiring control [16].

Meanwhile, external structures like state policy, legal frameworks, and health services exact structural violence by either failing to recognise *hi-fun* communities or by actively persecuting them. Dominant moral discourses draw lines between ‘us’ and ‘them’, distinguishing those deemed worthy of support from those cast as criminal or needing rehabilitation. Biomedical definitions of drug users as patients or addicts not only categorise but serve to allocate power and distribute resources. Recognising *hi-fun* participants as equal citizens would necessitate dismantling such frameworks and redistributing state resources, a challenge to entrenched systems that prioritise other populations. As a result, the state maintains its authority by rendering *hi-fun* invisible or illegitimate. Non-recognition, neglect, and resource denial function as a politics of control upheld by structural discourses, effectively erasing *hi-fun* participants and reinforcing inequitable distribution [35,46].

Supporting men engaged in *hi-fun* in Thailand requires critically examining state resource allocation and recognising *hi-fun* participants as citizens entitled to dignity and human rights. Findings from this study suggest that abstinence-based, compulsory treatment, and punitive legal-health models fail to meet the lived realities and well-being needs of *hi-fun* communities while also enacting structural violence [31]. Participants clearly expressed the need for access to post-use recovery resources (e.g., vitamins, detox herbs), clean injecting equipment, and stigma-free spaces. These priorities are consistent with previous calls for community-informed harm reduction approaches [5,11,47].

Although some harm reduction and recovery services do exist, they are largely concentrated in the private sector and remain financially accessible only to middle- and upper-middle-class Thais or affluent foreigners, or are research initiatives with capacity potentially constrained by funding [48,49]. The limited availability of comparable state-supported services addressing these needs may reflect not simply a resource gap but a deeper moral and ideological boundary in which sexualised drug use is cast as deviant rather than recognised as a legitimate public health issue. This moral framing limits the state’s willingness to adopt and invest in evidence-informed harm reduction strategies, thereby reinforcing structural inequities in access to healthcare.

These findings support community-driven harm reduction approaches that are grounded in local knowledge and the lived experiences of GBMSM engaged in *hi-fun* [50]. Several practical strategies could be implemented within Thailand’s current prohibition-oriented policy environment. These include strengthening peer-led education, supporting mutual aid networks among GBMSM engaged in *hi-fun*, providing safer drug-use information and clean injecting equipment, and improving access to non-judgemental recovery resources such as vitamins and detox herbs. Because *hi-fun* gatherings are often carefully planned in advance, opportunities also exist to integrate harm reduction into the preparation process through community outreach and pre-session education. These community-based approaches may be particularly feasible because they do not require immediate changes to the legal and moral systems that regulate sexuality and substance use in Thailand, although broader legal reform remains important. At the same time, broader efforts to reduce stigma towards people who use drugs remain essential, as stigma continues to be a major barrier to service access and development in Thailand. Consistent with this, participants highlighted the importance of non-judgemental support, acceptance, and respectful treatment from families, communities, and healthcare providers in helping them manage their drug use and maintain their well-being.

## Strengths and limitations

A key strength of this study lies in amplifying the voices of GBMSM with direct experience of *hi-fun*. The study also captures the diversity of local settings by covering three distinct research sites across Thailand. Moreover, participants represented a broad range of backgrounds in terms of country of birth, education and age, which allowed for insights into both similarities and differences across groups.

Nevertheless, this study has several limitations. Participant recruitment was primarily conducted through local NGOs, due to concerns about legal risks (see [4]). As a result, all participants were individuals who had access to health services provided by these NGOs. Consequently, the study lacks the perspectives of GBMSM who are unable to access such services, or who remain hidden from NGO support systems.

Focus group discussions tended to centre on normative understandings of *hi-fun* rather than personal, in-depth experiences. This limited the richness of data related to legal issues, such as arrest, incarceration, or engagement with rehabilitation programmes within focus groups. Participants may have felt uncomfortable sharing sensitive personal experiences in a group setting, which may have resulted in some surface-level reflections.

Finally, although the study intended to include both cisgender men and transgender men, all participants recruited were cisgender men. The absence of trans male participants may indicate contextual differences in *hi-fun* participation between these groups, which remains an important gap for future research.

## Conclusions

This research highlights the complexities that coexist within the *hi-fun* spaces of GBMSM in Thailand. *Hi-fun* is experienced as a sphere of freedom, emotional connection and relief from everyday stressors, offering opportunities to express aspects of oneself that are often constrained in everyday life. It also serves as a site for building networks and relationships that enhance participants’ sense of self-worth. However, *hi-fun* is also deeply connected with subcultural norms and broader economic and legal forces that shape and sometimes constrain these experiences.

Within this world of mixed pleasures, many participants voiced underlying concerns such as losing control over their substance use, which could affect their work and their mental health. They also described situations where diminished self-control left them vulnerable to violence, coercion, or threats. For participants, these experiences signalled that *hi-fun* was becoming problematic, not just a challenge or a risk. The possibility of arrest, prosecution, or compulsory treatment was seen as intensifying this further.

Ultimately, what GBMSM in the *hi-fun* context most need is not the punitive legislation and stigmatisation they currently face, but recognition of their humanity, genuine understanding, and meaningful support. This includes listening to their voices, creating environments free from discrimination, and ensuring access to resources that enable safer substance use. Most importantly, this requires reforms in laws and policies that reflect lived realities, acknowledge people who use substances as human beings with dignity, and provide opportunities for them to live stable and safe lives, free from marginalisation.
